# Cytokine profiling in serum-derived exosomes isolated by different methods

**DOI:** 10.1038/s41598-020-70584-z

**Published:** 2020-08-21

**Authors:** Hae Hyun Jung, Ji-Yeon Kim, Ji Eun Lim, Young-Hyuck Im

**Affiliations:** 1 Department of Health Sciences and Technology, Samsung Advanced Institute for Health Sciences and Technology, Sungkyunkwan University, Seoul, 06351 Korea; 2 Samsung Biomedical Research Institute, Samsung Medical Center, Seoul, 06351 Korea; 3 Division of Hematology-Oncology, Department of Medicine, Samsung Medical Center, Sungkyunkwan University School of Medicine, 81 Irwon-ro, Gangnam-gu, Seoul, 06351 Korea

**Keywords:** Biological techniques, Cancer, Biomarkers

## Abstract

Exosomes in blood play an important role in cell-to-cell signaling and are a novel source of biomarkers for the diagnosis and prognosis of diseases. Recently, evidence has accumulated that cytokines are released from encapsulated exosomes and are capable of eliciting biological effects upon contact with sensitive cells. However, there is currently limited information on exosome isolation methods for cytokine research. In this study, we evaluated three exosome isolation methods for their usability, yield, purity, and effectiveness in subsequent cytokine profiling. We found that ultracentrifugation (UC) and Exoquick (EQ), but not exoEasy, yielded appropriate exosome sizes, and EQ had higher exosome extraction efficiency than the other two methods. Although UC generated markedly fewer particles than EQ, it yielded a relatively high purity. Next, we performed a multiplex assay with the ProcartaPlex Immune Monitoring 65-Plex Panel to determine the feasibility of these methods for cytokine profiling. The results indicated significant differences among isolation methods when analyzing exosomal cytokine profiles. We further investigated the changes of exosomal cytokines according to breast cancer progression in triple-negative breast cancer. We found significantly decreased concentrations of MIP-3 alpha, IL-23, M-CSF, Eotaxin-3, BLC, SDF-1 alpha, IL-2R, MDC, FGF-2, IL-22, and IL-31 in exosomes from metastatic breast cancer (MBC) patients.

## Introduction

Bidirectional communication between cells and their microenvironment is critical for maintenance of the body in both physiological and pathological conditions. In addition to traditional soluble factors (chemokines, cytokines, growth factors), a growing body of evidence indicates that extracellular vesicles (EVs) play an important role in cell–cell communication^1^. Extracellular vesicles (EVs), which are highly heterogeneous, comprise exosomes, shed microvesicles (sMVs), and apoptotic bodies classified by their size, density, and origin^2^. Exosomes, small microvesicles of 30–150 nm with endosomal origin, are produced by most cells and are actively released by fusion of the microvesicular bodies with the plasma membrane^3,4^. They carry diverse and complex cargo molecules, such as nucleic acids (DNAs, RNAs), lipids, and proteins, and play an important role in intercellular communication by serving as a carrier for the transfer of these bioactive cargoes between cells. Particularly in cancer, exosomes can take part in the modification of the tumor microenvironment, favoring tumor progression and metastasis^5,6^. Moreover, exosomes are emerging as potential diagnostic, prognostic, and predictive biomarkers for disease status and treatment outcomes^7–9^.

Cytokines are small proteins secreted by cells that have a specific role in the interaction and communication between cells. Cytokines are widely recognized as crucial factors in cancer development and therapeutic resistance^10,11^. Several studies have reported that exosomes deliver cytokines such as TGF-β, TNF-α, IL-6, IL-8, and IL-10 to the recipient cells, leading to development and progression of cancer and drug resistance^12–16^. These studies suggest that exosome-associated cytokines (exosomal cytokines) could be novel biomarkers.

The isolation and characterization of pure exosomes is critical to the understanding of their biological mechanisms. Various methods based on different principles have been developed for exosome isolation. However, the most effective approach for exosome isolation is not yet well established. The recommendations in the guidelines of the International Society for Extracellular Vesicles (ISEV) are not definite and leave extracellular vesicle (EV) isolation to the investigators’ discretion^17^. No gold standard for exosome isolation exists and exosome isolation from serum or plasma is especially problematic because of the small volume available, its high viscosity, and its high concentration of proteins and lipoproteins^18^. For these reasons, it is very important to find a suitable exosome isolation method and identify its potential for analysis of exosomal cytokine biomarkers.

Here, we isolated exosomes from pooled serum of breast cancer patients (N = 30) using three different techniques: ultracentrifugation and two commercial kits, exoEasy (EE; membrane affinity spin column method) and ExoQuick (EQ; polymer-based precipitation). We characterized the size and yield of enriched exosomes using Nanoparticle Tracking Analysis (NTA) and ExoView, then determined the purity of the exosomes using their protein concentration by silver stain and Western blot. We then analyzed their cytokine content using a multiplex assay with the ProcartaPlex Immune Monitoring 65-Plex Panel. We further evaluated exosomal cytokines as potential biomarkers in breast cancer. We compared the exosomal and serum cytokine changes between ductal carcinoma in situ (DCIS), early breast cancer (EBC), and advanced metastatic breast cancer (MBC) with the triple-negative subtype.

## Results

### Characterization of isolated exosomes

**Figure 1:**
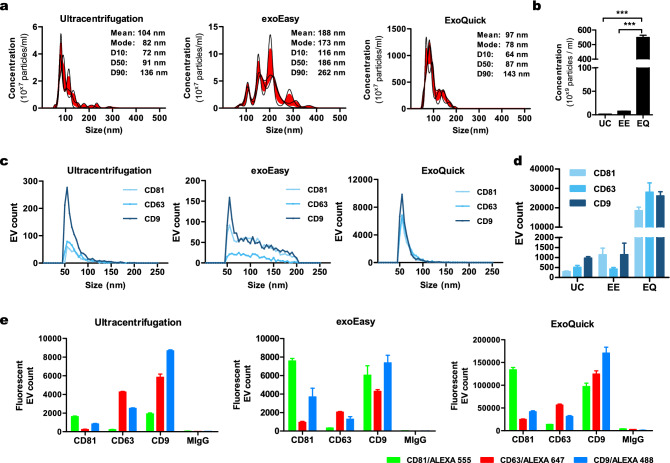
Characterization of exosomes using NTA and SP-IRIS immunophenotypic analysis. NTA was done to measure particle distribution (**a**) and the total number of particles (**b**) isolated using ultracentrifugation, exoEasy, and ExoQuick. The calculated size distribution is depicted as a mean (black line) with standard error (red shaded area). The mean and mode size of particles are shown for each graph. SP-IRIS immunophenotypic analysis by the multiplexed microarray ExoView was done to measure particle distribution (**c**) and the number (**d**) of CD81, CD63, and CD9-positive exosomes. Exosome samples were incubated with microarray chips coated with the indicated antibodies. Images of scattered light were taken and analyzed regarding particle distribution and total exosome number (captured exosomes). Co-expressions of exosome markers (**e**) were measured by probing captured EVs with the indicated secondary fluorescence-labeled antibody. *UC* ultracentrifugation, *EE* exoEasy, *EQ* ExoQuick.

Exosomes were prepared from the pooled serum using three methods: differential ultracentrifugation (UC), exoEasy kit (EE), and ExoQuick solution (EQ). To evaluate the size distribution, exosomes isolated from different methods were subjected to NTA, which revealed that the size distribution curve of the EE method was broader than those of the UC and EQ methods (Fig. 1a). All the exosome preparations from the UC and EQ methods showed the accepted size range (30–150 nm), but not those from the EE method. The EE method exhibited the largest mode size compared to the UC and EQ methods (mean ± SD; EE 172.6 ± 25.7 nm > UC 81.9 ± 6.5 > EQ 77.7 ± 8.6 nm). Further analysis of the concentration of exosomes by NTA revealed that the EQ method is appropriate for isolation of larger numbers of particles (5.5 × 10^11^ particles/mL), followed by the EE method (7.6 × 10^9^ particles/mL). Enrichment of exosomes by UC yielded the lowest concentration (1.5 × 10^9^ particles/mL) (Fig. 1b).

To obtain more elaborate results, exosome fractions were analyzed by SP-IRIS using the ExoView platform in order to quantify exosomes depending on their expression of marker proteins. Exosomes were captured by antibodies targeting the common exosome markers CD9, CD63, and CD81 and subsequently imaged and quantified. Overall, the NTA results correlated well with the ExoView results, but the exosome size measured by ExoView was smaller than the value measured by NTA. Similar to NTA, the exosome size distribution of the EE method was broader and more heterogeneous than those of the UC and EQ methods (Fig. 1c). Captured exosome counts were highest with the EQ method. More exosomes were captured by CD9 than by CD63 and CD81 with the UC method, and less exosomes were captured by CD63 than by CD9 and CD81 with the EE method. With the EQ method, the numbers of exosomes captured by the three markers were similar (Fig. 1d). ExoView allowed analysis of the differential co-expression of exosome markers on a single exosome basis by probing captured exosomes with a secondary fluorescence-labeled antibody (Supplementary Fig. 1). ExoView analysis showed that CD81-captured exosomes had low CD63 co-expression, while exosomes co-expressing CD63 and CD9 were relatively high in all three methods (Fig. 1e).

In summary, among the three methods, UC and EQ were most appropriate for selecting exosome size, and EQ showed the highest exosome extraction efficiency when isolating a large number of particles. We confirmed that exosome size, exosome extraction efficiency, and expression of exosome marker proteins differ according to extraction method.

### Exosome purity

**Figure 2:**
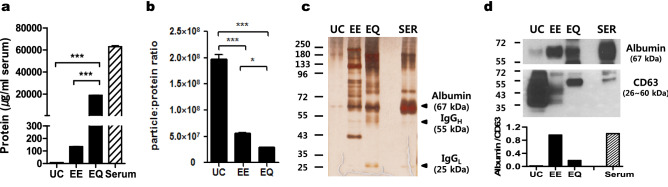
Purity measurements of exosomes isolated using different methods. (**a**) Protein concentration of the intact isolated exosomes was determined by bicinchoninic acid (BCA) assay. (**b**) Comparison of exosome purity index (concentration of particles expressed as a ratio per µg of protein) (**c**) Silver staining of the exosome fractions. The exosome fraction (10^8^ particles) was run on SDS-PAGE, and the proteins were stained by silver staining. (**d**) Western blotting analysis for CD63 and albumin in serum and exosomes isolated using three methods. The band density of albumin and CD63 was quantified with ImageJ. The data represent means ± SD of three independent experiments. *p < 0.05; **p < 0.01; ***p < 0.001. *UC* ultracentrifugation, *EE* exoEasy, *EQ* ExoQuick, *SER* Serum, *IgG*_*H*_ immunoglobulin G heavy chain, *IgG*_*L*_ immunoglobulin G light chain.

We measured the amount of protein present in the exosome samples to evaluate their purity. EQ yielded the highest protein content (18,871 ± 147 μg/mL), followed by EE (136 ± 1 μg /mL). The amount of protein determined in exosome fractions obtained by UC was the lowest (8 ± 0.2 μg /mL) (Fig. 2a). For measurement of particle purity, the particle-protein ratio^19^ was used. The ratio was significantly lower for EE and EQ than for UC, indicating co-isolation of serum proteins in EE and EQ (Fig. 2b). Protein yield, expressed as relative protein amount per 10^8^ particles, was 3.6- and 6.8-fold respectively higher in exosome preparations from EE and EQ, respectively, compared to UC (data not shown). These samples were tested for protein distribution by silver stain. Albumin, the most abundant plasma protein, was readily detectable in EE and EQ preparations (Fig. 2c). To further assess the purity, western blot analysis was performed with equal protein loading to allow direct evaluation of the exosome sample purity by comparing the enrichment of proteins regarded as an exosomal marker (CD63) and albumin. Accordingly, we detected a dramatic enrichment of CD63 in exosomes isolated by UC but not from EE and EQ (Fig. 2d). The high protein content and the low particle-protein ratio together with the low levels of exosome markers isolated from the EQ and EE methods suggest co-isolation of contaminating serum proteins.

### Profiles of cytokines from serum and exosomes

**Figure 3:**
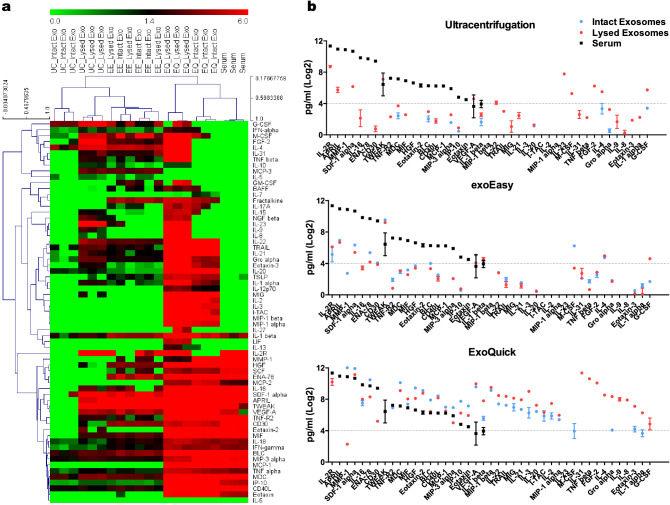
Comparison of cytokine expression in exosomes isolated using three different methods. Cytokines were detected from serum, intact EV, and lysed EV by multiplex assay. (**a**) Heatmap showing unsupervised hierarchical clustering of samples, generated with Multi-Experiment Viewer (MeV v4.9). (**b**) Scatter plot showing mean expression difference for three different methods. Each point represents the mean of the results for triplicate samples. Graphs were generated using GraphPad Prism 5.

To examine the variations in cytokine expression with different exosome isolation methods, we performed a multiplex assay using the ProcartaPlex Immune Monitoring 65-Plex Panel. Intact exosomes, lysed exosomes, and serum were used for the assay. The amounts of cytokines were calculated by multiplication of the ratio of starting serum volume. The heatmap showed that cytokine profiling was different according to the isolation method. In the UC and EQ methods, cytokines were released more from lysed exosomes than from intact exosomes, whereas in the EE method, relatively small amounts of cytokines were released from both types of exosomes in similar amounts (Fig. 3a, Supplementary Table 1).

Analysis of individual cytokines in exosomes isolated by UC revealed that 12 cytokines, IL-2R, APRIL, SDF-1 alpha, TWEAK, MDC, VEGF-A, IL-22, IL-23, M-CSF, FGF-2, IL-4, and G-CSF, were found in greater levels in lysed exosomes than in intact exosomes. In the EE method, cytokine levels, except TWEAK and APRIL, were very low in both types of exosome. Analysis of cytokines in exosomes isolated by EQ revealed that 13 cytokines, IL-23, M-CSF, IL-31, TNF beta, IL-2R, FGF-2, IL-4, Gro-alpha, IL-9, IL-8, Eotaxin-3, IL-1 alpha, and G-CSF, were found in lysed exosomes. However, other cytokines were detected in intact as well as lysed exosomes, indicating co-isolation of blood proteins (Fig. 3b).

We also compared exosomal cytokine profiling using data normalized by the number of isolated exosomes using different exosome isolation methods. The heatmap showed that cytokine expressions per exosomal particle were the highest in the UC method (Supplementary Fig. 2). Based on the above results, we used the UC method in our further studies to avoid confounding results regarding exosome biomarker candidates.

### Changes of exosome-associated cytokines according to breast cancer types and stages in triple-negative breast cancer

Next, we investigated the possibility of exosome-associated cytokines as prognostic biomarkers in triple-negative breast cancer (TNBC), the most aggressive breast cancer subtype. TNBC is defined by no expression of estrogen receptor (ER) and progesterone receptor (PR), and no amplification or overexpression of HER2^20^. Recent research has demonstrated that TNBC has higher immunogenicity than other subtypes, as it has a significant number of genetic mutations and robust tumor immune cell infiltration^21^.

**Figure 4:**
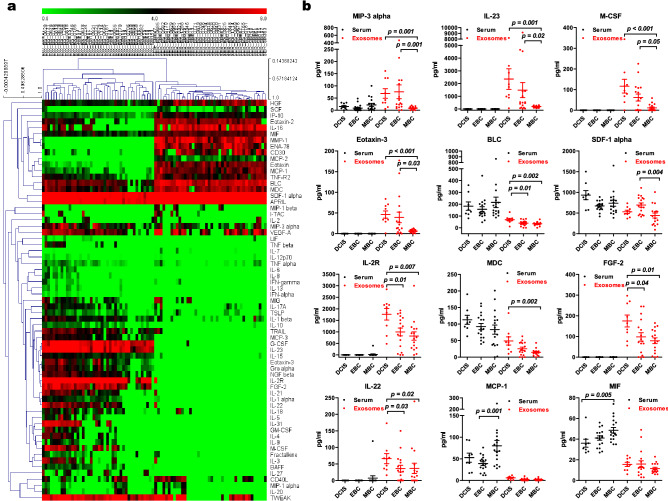
Serum and exosomal cytokine expression in patients with DCIS, EBC, and MBC. Exosomes were isolated from 250 µL of patients’ sera by serial ultracentrifugation. Cytokines were detected from serum and lysed exosomes by a multiplex assay. (**a**) Heatmap showing unsupervised hierarchical clustering of samples, generated with Multi-Experiment Viewer (MeV v4.9). (**b**) Scatter plots show the comparison of serum and exosomal cytokine concentrations in patients with DCIS (n = 9), early breast cancer (n = 17), and metastatic breast cancer (n = 17). The statistical analysis was performed by Mann‐Whitney-U test analysis. *p* < 0.05 was considered statistically significant. Graphs were generated using GraphPad Prism 5.

**Figure 5:**
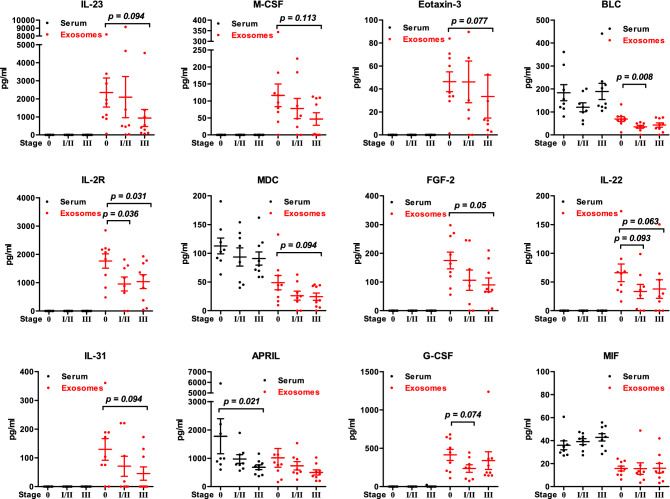
Serum and exosomal cytokine expression according to each stage in patients with DCIS and EBC. Exosomes were isolated from 250 µL of patients’sera by serial ultracentrifugation. Cytokines were detected in serum and lysed exosomes by multiplex assay. Scatter plots compare serum and exosomal cytokine concentrations in patients with DCIS (stage 0, n = 9), early-stage breast cancer (stage I/II, n = 8), and advanced-stage breast cancer (stage III, n = 9). The statistical analysis was performed by Mann‐Whitney-U test analysis. *p* < 0.05 was considered statistically significant.

Exosomes were isolated from serum obtained from patients with ductal carcinoma in situ (DCIS, n = 9), early breast cancer (EBC, n = 17), and metastatic breast cancer (MBC, n = 17) using the UC method. The clinical information of the patients is summarized in Table 1. Serum and exosomal cytokine levels were determined by a multiplex assay using the ProcartaPlex Immune Monitoring 65-Plex Panel. The values of cytokines were subjected to unsupervised-hierarchical cluster analysis, and the result showed that the serum and lysed exosome samples were accurately distinguished based on the samples (Fig. 4a). The Kruskal–Wallis test was used to statistically analyze the values of cytokines. We obtained 13 cytokines differentially expressed among DCIS, EBC, and MBC (Table 2). We found significantly decreased concentrations of MIP-3 alpha, IL-23, M-CSF, Eotaxin-3, BLC, SDF-1 alpha, IL-2R, MDC, FGF-2, IL-22, and IL-31 in MBC patients’ exosomes. In contrast, the concentrations of MCP-1 and MIF increased in MBC patients' serum (Fig. 4b). Furthermore, we analyzed the variation in serum and exosomal cytokine expression according to stages. We divided patients into a DCIS group (stage 0, n = 9), an early-stage group (stage I/II, n = 8), and an advanced-stage group (stage III, n = 9). Similar to Fig. 4, exosomal cytokine concentrations tended to decrease according to the disease stage of the patient (Fig. 5). However, the results were not statistically significant, possibly due to our small sample size.

**Table 1:** Patient characteristics.

		DCIS	EBC	MBC
		n = 9	n = 17	n = 17
**Age**				
Median		42.50	53.00	42.50
Range		30–76	35–72	33–54
**Histology**				
DCIS		9	–	–
IDC		–	17	14
Others		–	0	3
**Tumor size**				
Tis		9	–	–
T1		–	7	7
T2		–	5	4
T3		–	4	3
T4		–	1	1
Unknown		–	0	2
**Lymph nodes**				
N0		9	7	5
N1		–	1	2
N2		–	3	3
N3		–	6	5
Unknown		–	0	2
**Nuclear grade**				
1		3	0	0
2		3	5	7
3		3	12	8
Unknown		0	0	2
**Histology grade**				
1		–	1	1
2		–	4	10
3		–	12	4
Unknown		–	0	2
**Stage**				
0		9	–	–
I		–	6	5
II		–	2	2
III		–	9	8
IV		–	0	2
**Adjuvant chemotherapy**				
Yes		–	16	13
No		–	1	4
**Adjuvant radiotherapy**				
Yes		7	15	14
No		2	2	3
**Overall survival**				
Alive		9	9	4
Dead		-	8	13

**Table 2:** Summary of the statistical significance of all group comparisons.

	DCIS serum (n = 9) Mean ± SE	EBC serum (n = 17) Mean ± SE	MBC serum (n = 17) Mean ± SE	*p *value	DCIS exosomes (n = 9) Mean ± SE	EBC exosomes (n = 17) Mean ± SE	MBC exosomes (n = 17) Mean ± SE	*p *value
MIP-3 alpha	15 ± 4.5	8 ± 3.3	22 ± 7	0.118	75 ± 20.5	75 ± 28.3	8 ± 1.5	0.001
IL-23	–	–	–	1	2,506 ± 898.1	1,482 ± 590.1	185 ± 19.5	0.002
M-CSF	–	–	–	1	122 ± 37.1	61 ± 16.9	11 ± 4.5	0.002
Eotaxin-3	–	–	–	1	47 ± 9.6	39 ± 12.7	6 ± 0.9	0.003
BLC	183 ± 35	156 ± 21.9	217 ± 47.2	0.500	69 ± 12	39 ± 5.7	33 ± 4.1	0.008
SDF-1 alpha	931 ± 110.3	672 ± 29.2	752 ± 73.6	0.090	536 ± 43.7	692 ± 51.2	439 ± 61	0.010
IL-2R	–	–	25 ± 25.1	0.479	1825 ± 277	998 ± 168.9	819 ± 181.8	0.012
MDC	112 ± 13.6	92 ± 9.2	84 ± 14.6	0.279	51 ± 13.6	25 ± 4.8	14 ± 2.5	0.012
FGF-2	–	–	–	1	181 ± 31.8	97 ± 20.2	79 ± 14.1	0.036
IL-22	–	–	7 ± 7.4	0.479	68 ± 17.1	35 ± 9.9	36 ± 15.5	0.041
IL-31	–	–	–	1	145 ± 38.5	57 ± 20.2	35 ± 14.9	0.044
APRIL	1777 ± 619.7	819 ± 89.9	876 ± 116.3	0.103	1,071 ± 370.2	608 ± 86.6	457 ± 98.9	0.056
G-CSF	0 ± 0	1 ± 1.2	0 ± 0	0.479	439 ± 76.5	290 ± 65	217 ± 29.3	0.057
MCP-1	52 ± 10.5	38 ± 4.4	80 ± 13.3	0.006	5 ± 1	2 ± 0.6	1 ± 0.5	0.010
MIF	36 ± 3.8	41 ± 2.1	49 ± 2.7	0.013	15 ± 2.3	15 ± 3.1	11 ± 1.2	0.304

Serum and exosomal cytokines between different groups were analyzed using the Kruskal–Wallis test. *P* < 0.05 was considered statistically significant. The unit of measurement is in pg/mL.

*DCIS* ductal carcinoma in situ, *EBC* early breast cancer, *MBC* metastatic breast cancer.

## Discussion

As the potential for clinical applications of exosomes increases, it is crucial to optimize the isolation method in terms of purity, yield, and reproducibility. Despite the recent development of several isolation methods for exosomes, there is still no standardization and new methods are often used without well-organized comparative analysis.

Several studies have attempted to compare the effect of various exosome isolation techniques on downstream analyses. Some groups have conducted comparative studies on different isolation methods regarding their effects on subsequent RNA^22–26^ and proteomic^27,28^ analysis. However, there is limited information regarding proper isolation methods for exosomal cytokine analysis. Although Macías et al.^29^ evaluated six commercial exosome kits, including EQ and EE, for exosomal cytokines in serum, they evaluated only three cytokines (S100A9, CXCL5, and CXCL12) and the methods they used did not include UC, the traditional exosome isolation method.

In this study, we provided a comparative evaluation of three exosome isolation methods for their size, yield, purity, and effects in subsequent cytokine profiling. We isolated exosomes from serum using three different techniques: ultracentrifugation (the most common EV isolation methods), exoEasy (EE; membrane affinity spin column method)^30^ and ExoQuick (EQ; polymer-based precipitation). The size-exclusion chromatography method was omitted as it requires a second step to concentrate samples, although it can isolate more purified exosomes^31^.

We characterized the size, yield, and exosome markers of enriched exosomes using Nanoparticle Tracking Analysis (NTA) and ExoView. We found that all methods were appropriate to isolate exosomes, as illustrated by ExoView for exosome markers. All the exosome preparations from the UC and EQ methods showed the accepted size range, but not those from the EE method. The EE-isolated exosomes were a heterogenous mixture of particles with a larger diameter and broader size range than the UC- and EQ-isolated exosomes. This heterogeneity has been described by other groups^32^. The EQ method shows the best exosome extraction efficiency. These results are consistent with previous reports showing that the concentration of exosomes with EQ is more efficient than with UC^25,33^. To assess the presence of impurities, we used the particle to protein ratio and albumin (frequent contaminant of exosomes isolated from human serum). Albumin impurity was ubiquitous for all methods. The UC method provided the highest purity, suggesting less protein contamination with this method. However, we did not test lipoprotein contamination, which has a size in the range of exosomes^18^.

After confirming the characteristics of exosomes, we analyzed their variation in cytokine profiles according to isolation method by multiplex assay using the ProcartaPlex Immune Monitoring 65-Plex Panel. We applied intact exosomes and serum, as well as lysed exosomes, to identify serum protein contamination. We observed significant differences in the exosomal cytokine profile depending on the isolation method and preparations. Many cytokines were detected by the EQ method, but its expression tendency was similar to that of serum and simultaneously expressed in intact exosomes, indicating the possibility of co-elution of serum cytokines. The UC method, on the other hand, is thought to be suitable for biomarker studies without confusing results because cytokines were identified mainly from the lysed form. There is a possibility that cytokines on the surface of intact exosomes could be detected as well as serum cytokines^34^. A limitation of this study is that we cannot distinguish whether the cytokines were detected from intact exosomes, including cytokines bound on the exosome surface. However, since intact exosomes showed relatively low cytokine expression, we concluded that the UC method is the most appropriate method when isolating exosomes from blood. The UC method, however, needs to be tightly controlled because the yield varies with several parameters such as rotor type, sample viscosity, and centrifugation time^35^.

Next, we investigated the changes of exosomal cytokines according to breast cancer types and stages in triple-negative breast cancer (TNBC). Several studies have reported that exosome biomarkers (such as CD44, CD47, CXCR4, Del-1, HER2, and KDR) can be used for early diagnosis and prognosis of breast cancer patients^36^. However, there are no studies regarding exosomal cytokines as biomarkers in breast cancer. Cytokine expression was evaluated in serum and exosomes isolated from ductal carcinoma in situ (DCIS), early breast cancer (EBC), and advanced metastatic breast cancer (MBC) with the triple-negative subtype. The exosomal cytokine level tended to decrease with the stage of disease (DCIS > EBC > MBC). MBC patients’ exosomes had a low concentration of MIP-3 alpha, IL-23, M-CSF, Eotaxin-3, BLC, SDF-1 alpha, IL-2R, MDC, FGF-2, IL-22, and IL-31, suggesting that metastatic disease may be associated with immune suppression related to low exosomal cytokines. These results are consistent with previous reports that the levels of the cytokines IFN-γ, IL-10, and IL-3 were decreased in plasma exosomes from grade 4 GBM (glioblastoma multiforme) patients compared to normal donors^37^. Unlike exosomes, MBC patients' serum had a high concentration of MCP-1 and MIF, suggesting that high levels of MCP-1 and MIF play an important role in metastasis. Our findings showed the potential role of exosomal cytokines as a blood-based biomarker in more aggressive tumors. Further large-scale studies are needed to prove the usefulness of exosomal-derived cytokines as biomarkers in clinical practice.

In this study, we demonstrated that the exosome isolation method greatly affects the cytokine profile. We also found that the UC method is more suitable than the EE and EQ methods for exosomal cytokine studies. Our results could serve as a guide when selecting exosome isolation methods to find reliable biomarkers in serum. Furthermore, we showed that 11 exosomal cytokines were decreased according to the progression of TNBC. This study is, to the best of our knowledge, the first study that proves the clinical significance of exosomal cytokines in breast cancer.

## Material and methods

### Patients and blood samples

All patients were diagnosed with breast cancer at the Samsung Medical Center between 2008 and 2010. Pooled serum samples from breast cancer patients (N = 30) were provided by Samsung Medical Center BioBank. Forty-three samples were analyzed in this study, including samples from patients diagnosed with DCIS (n = 9), EBC (n = 17), and MBC (n = 17) with triple-negative subtype. DCIS and EBC samples were collected prior to surgical resection and chemotherapy and were provided by Samsung Medical Center BioBank. Patients with MBC received palliative chemotherapy with a median of 1.4 regimens (range, 1–3 regimens) before their enrollment. Blood samples were incubated for 30 min in room temperature for clotting, then centrifuged for 20 min at 3,000 rpm. Serum was isolated and stored at − 80 °C until analysis. All patients provided written informed consent to access their blood and review their medical records. The study was approved by the ethics committee of Samsung Medical Center (IRB No: 2010-08-077, 2017-12-068) and was conducted in accordance to the Declaration of Helsinki. All experiments were performed in accordance with relevant guidelines and regulations.

### Exosome isolation from serum

Exosomes were isolated from serum samples using differential centrifugation and two commercial kits: ExoQuick Serum Exosome Precipitation Solution (System Biosciences, Palo Alto, CA, USA) and exoEasy kit (Qiagen GmbH, Hilden, Germany). Serum samples were centrifuged at 2,000×*g* for 10 min and 10,000×*g* for 30 min at 4 °C to thoroughly remove cellular debris. Next, supernatants were filtered through a 0.2 μm pore size filter to exclude particles > 200 nm in diameter. For exosome isolation by differential ultracentrifugation, the filtered supernatant was collected into ultracentrifuge tubes and centrifuged in an Optima MAX-XP ultracentrifuge with an MLA-130 rotor (Beckman Coulter, Jersey City, NJ, USA) at 100,000 g for 1 h at 4 °C. The pellets were washed with PBS, ultracentrifuged again, and resuspended in PBS. For exosome isolation by ExoQuick and exoEasy, the two commercial kits were used according to the manufacturers’ instructions. Exosome preparations were conserved at − 80 °C for later use.

### Nanoparticle tracking analysis (NTA)

The size distribution and concentration of the serum-derived exosomes were analyzed by NTA using a NanoSight model NS300 equipped with a red laser and a SCMOS camera (Malvern Instruments, Malvern, UK). The samples were diluted in 1 ml PBS and mixed well, and the diluted samples were then injected into the laser chamber. The following settings were used for data acquisition: camera level 14, acquisition time 30 s, and detection threshold 3. Data analysis was performed with the NTA v3.2 software. Three recordings were performed for each sample.

### Nanoview analysis

Exosomes were detected using the ExoView Tetraspanin chip (ExoView, Boston, MA, USA) arrayed with antibodies against proteins CD81, CD63, and CD9. Mouse IgG1 was used as a negative control. Thirty-five µL of sample was dropped onto the chip surface and incubated overnight. After washing, the chips were treated with ExoView Tetraspanin Labelling ABs (EV-TC-AB-01), including CD9/ALEXA 488, CD81/ALEXA 555, and CD63/ALEXA 647, for co-localization tests in order to characterize the sub-populations on the surface of exosomes. The chips were then imaged with the ExoView R100 reader (ExoView) through the Single Particle Interferometric Reflectance Imaging Sensor (SP-IRIS) technology using the ExoScan v0.998 (ExoView) acquisition software. The data was analyzed using ExoViewer v0.998 with sizing thresholds set from 50 to 200 nm diameter.

### Protein isolation and quantification

Intact exosomes resuspended in PBS were lysed with 10 × RIPA buffer (Cell Signaling Technology, Danvers, MA, USA). Protein quantification of the lysed exosomes was performed using the Micro BCA Protein Assay Reagent Kit (Thermo Scientific, Waltham, MA, USA). The purity of exosome preparations was determined by calculating the ratio between particle number, determined by NTA, and protein concentration measured through Micro BCA assay. For silver staining, proteins were separated on 10% SDS-PAGE gels and visualized using a Pierce silver stain kit per the manufacturer’s instructions.

### Western blot

Total exosome proteins were isolated with RIPA buffer (Cell Signaling Technology). 10 µg of proteins per sample were separated by a NuPAGE 4–12% Bis–Tris gel (Invitrogen, Waltham, MA, USA) and transferred onto a PVDF membrane (Merck Millipore, Burlington, MA, USA). Membranes were blocked in 5% Bovine Serum Albumin in TBS-T for 2 h at room temperature and then incubated with primary antibodies against CD63 (Santa Cruz Biotechnology, Santa Cruz, CA, USA) and albumin (Cell Signaling Technology) overnight at 4 °C with gentle rocking. Membranes were washed, and HRP-conjugated antibodies were added for 1 h at room temperature. Detection was performed using enhanced chemiluminescence (ECL) reagents (Invitrogen) according to the manufacturer’s guidelines.

### Multiplex immunoassay

Intact exosomes, lysed exosomes, and serum samples were collected and stored at − 80 °C until analysis. Sample aliquots had not been previously thawed before use in the multiplex chemokine assay. We measured the levels of APRIL, BAFF, BLC, CD30, CD40L, ENA-78, Eotaxin, Eotaxin-2, Eotaxin-3, FGF-2, Fractalkine, G-CSF, GM-CSF, GROα, HGF, IFN-α, IFN-γ, IL-10, IL-12p70, IL-13, IL-15, IL-16, IL-17A, IL-18, IL-1α, IL-1β, IL-2, IL-20, IL-21, IL-22, IL-23, IL-27, IL-2R, IL-3, IL-31, IL-4, IL-5, IL-6, IL-7, IL-8, IL-9, IP-10, I-TAC, LIF, MCP-1, MCP-2, MCP-3, M-CSF, MDC, MIF, MIG, MIP1α, MIP-1β, MIP-3α, MMP-1, NGF beta, SCF, SDF-1α, TNF-β, TNF-α, TNF-R2, TRAIL, TSLP, TWEAK, and VEGF-A with the ProcartaPlex Human Immune Monitoring 65-Plex Panel (Invitrogen) using the Bio-Plex200 (Bio-Rad Laboratories, Hercules, CA, USA) according to the manufacturer’s instructions. Full names of cytokines are presented in Supplementary Table 2.

### Statistical analysis

All statistical analyses were performed using SPSS 20.0 software (IBM, USA), and the graphs were generated using GraphPad Prism 5.0 (GraphPad Software, San Diego, CA, USA). The comparisons between multiple groups were determined by analysis of variance (ANOVA) for parametric data and the Kruskal–Wallis test for nonparametric data. A value of *P* < 0.05 was considered statistically significant.

## Supplementary information

Supplementary Information.
