# Supplementary material for: Cytokine profiling in serum-derived exosomes isolated by different methods
Source: Sci Rep. 2020 Aug 21;10:14069. doi: 10.1038/s41598-020-70584-z (PMC7442638; doi:10.1038/s41598-020-70584-z)
Supplement: Supplementary file 1 — Supplementary Information. [file 41598_2020_70584_MOESM1_ESM.pdf]

Supplementary information

Cytokine Profiling in Serum-Derived Exosomes Isolated by Different Methods

Hae Hyun Jung[1], Ji-Yeon Kim[2,3], Ji Eun Lim[2], and Young-Hyuck Im[1,2,3*]

[1] Department of Health Sciences and Technology, Samsung Advanced Institute for Health Sciences and Technology, Sungkyunkwan University, Seoul, 06351, Korea

[2] Samsung Biomedical Research Institute, Samsung Medical Center, Seoul, 06351, Korea

[3] Division of Hematology-Oncology, Department of Medicine, Samsung Medical Center Sungkyunkwan University School of Medicine, Seoul, 06351, Korea

Corresponding authors: Young-Hyuck Im, MD, PhD, Division of Hematology-Oncology, Department of Medicine, Samsung Medical Center, Sungkyunkwan University School of Medicine, 81 Irwon-ro, Gangnam-gu, Seoul 06351, Korea. Phone: 82-2-3410-3445; Fax: +82-2-3410-1754; E-mail: yh00.im@samsung.com

Supplementary Figure 1

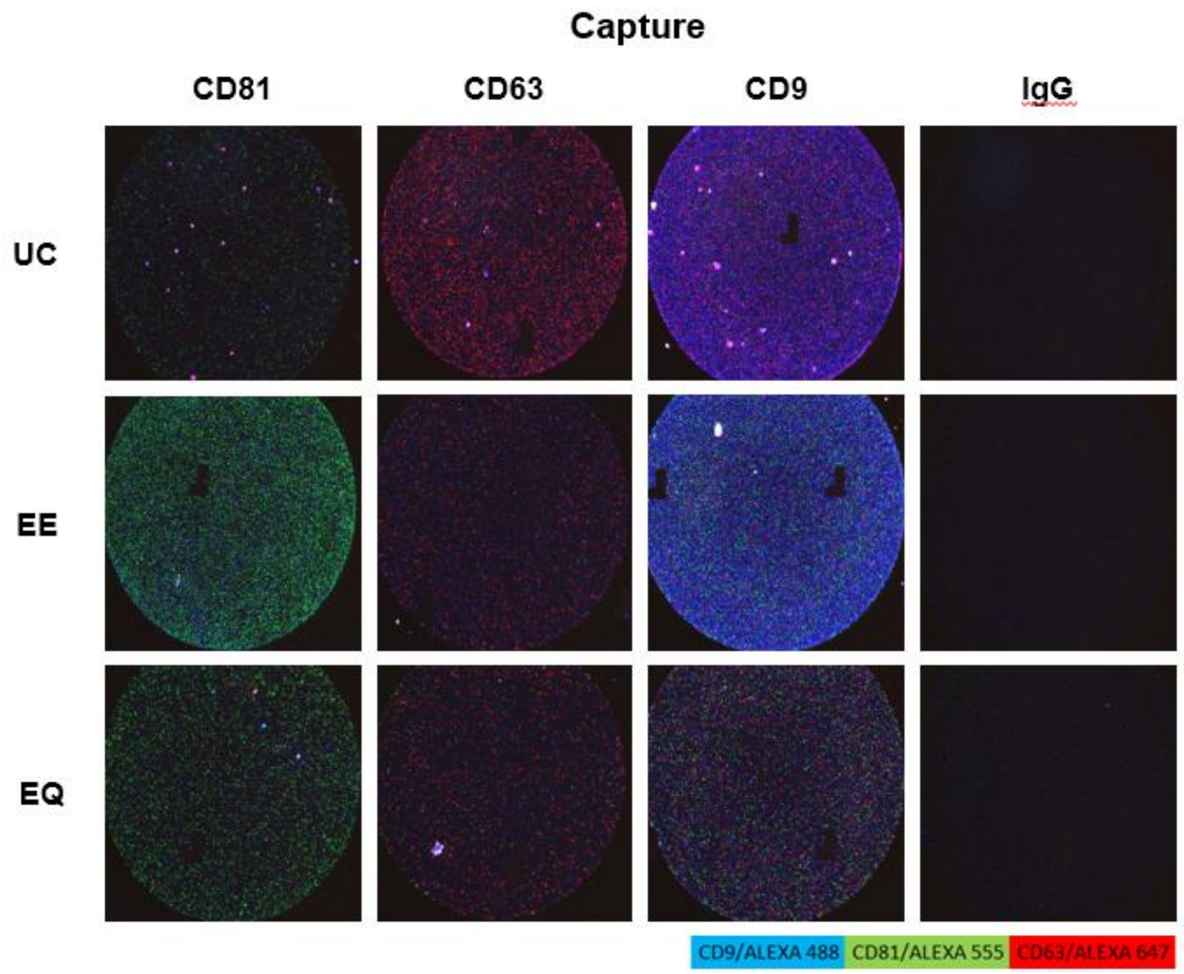

Supplementary Figure 1. Fluorescence image of ExoView indicating co-expression of exosome markers. Exosome samples were incubated with microarray chips coated with the indicated antibodies. Co-expression of exosome markers was measured by probing captured EVs with the indicated secondary fluorescence-labeled antibody. Abbreviations: UC, ultracentrifugation; EE, exoEasy; EQ, ExoQuick.

Supplementary Figure 2

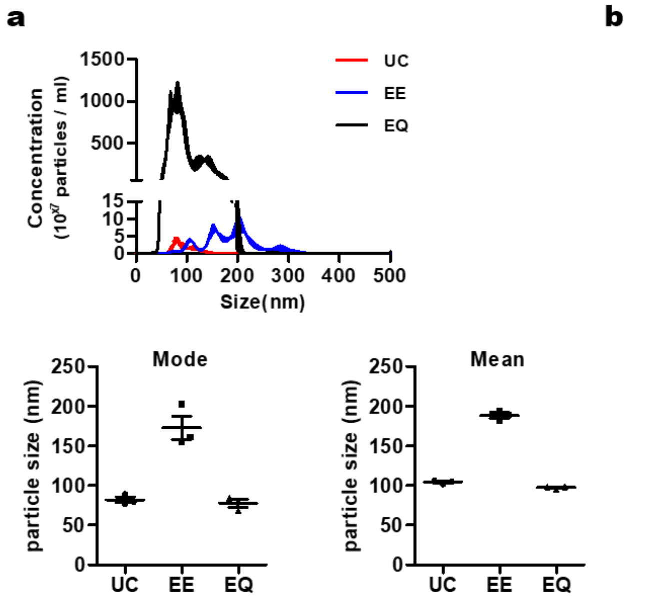

	UC	EE	EQ
Particle concentration (particles/ml)	1.5E+09 ± 1.2E+08	7.6E+09 ± 5.4E+08	5.5E+11 ± 2.6E+10
fold	1.0	4.9	358.0
Particle size-mode (nm)	81.9 ± 6.5	172.6 ± 25.7	77.7 ± 8.6
Particle size-mean (nm)	104.2 ± 2.5	188.1 ± 6.5	97.2 ± 1.9

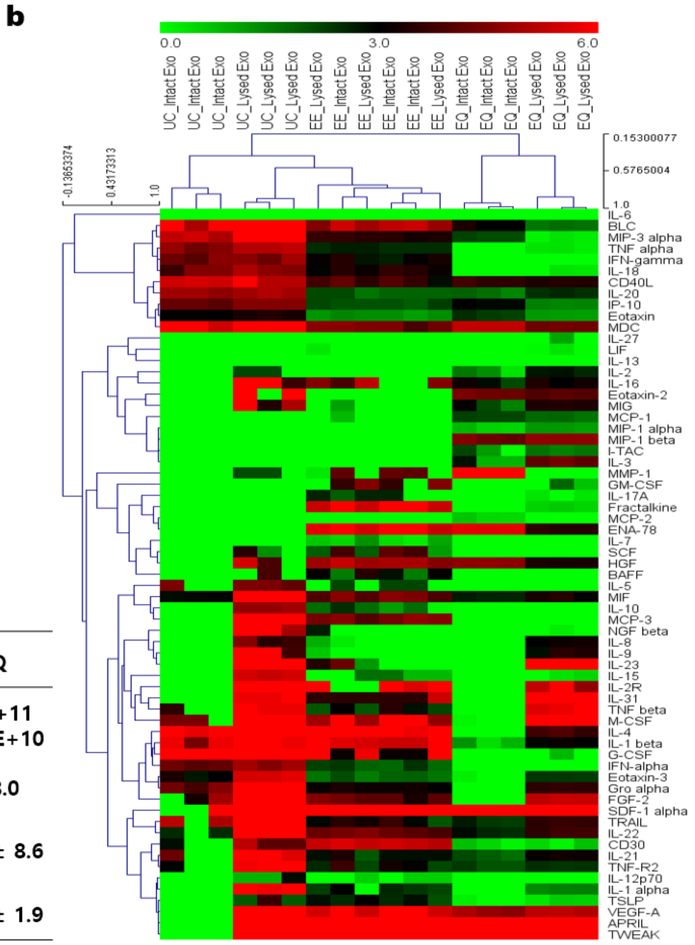

Supplementary Figure 2. Comparison of cytokine profile normalized by the number of isolated exosomes using three different methods. (a) NTA was done to measure particle distribution, particle size, and the total number of particles isolated using ultracentrifugation, exoEasy, and ExoQuick. (b) Cytokines were detected from serum, intact EV, and lysed EV by multiplex assay. The amounts of cytokines were normalized by cytokine expressions per 1.0.E + 10 exosomal particles. Heatmap showed unsupervised hierarchical clustering of samples and, generated with Multi-Experiment Viewer (MeV v4.9). Abbreviations: UC, ultracentrifugation; EE, exoEasy; EQ, ExoQuick.

Supplementary Table 1. Cytokine concentration according to isolation method and preparation. The unit of measurement is in pg/mL. Data are presented as the mean ± SE (n = 3).

Analyte	UC_Intact Exosome	UC_Lysed Exosome	EE_Intact Exosome	EE_Lysed Exosome	EQ_Intact Exosome	EQ_Lysed Exosome	Serum
APRIL	-	54.9 ± 13.7	123.5 ± 8.2	101.4 ± 11	4231.5 ± 163.1	5914.5 ± 198	1928.8 ± 52.9
BAFF	-	0.4 ± 0.4	1.6 ± 0.5	3.5 ± 0.3	2.7 ± 2.7	9.8 ± 2.3	-
BLC	4.3 ± 0.9	8 ± 0.5	15.7 ± 0.6	10.3 ± 0.7	242.7 ± 18.1	86.7 ± 2	77.2 ± 3.2
CD30	0.2 ± 0.2	1.8 ± 0.5	16.9 ± 0.9	14.8 ± 0.5	37.5 ± 4.5	37.5 ± 4.5	676.4 ± 21.6
CD40L	3.4 ± 0.1	3.5 ± 0.8	5.9 ± 0.6	4.1 ± 0.7	344.8 ± 18.8	292 ± 5.7	75.6 ± 3
ENA-78	-	-	41.5 ± 1.2	17.9 ± 1.3	1410.6 ± 17.6	312.1 ± 7.5	849.5 ± 34.1
Eotaxin	-	0.8	0.9 ± 0.1	0.9	137.8 ± 7.7	61.1 ± 0.7	22.9 ± 1.3
Eotaxin-2	-	5.4 ± 3	-	-	554.7 ± 19.9	437.6 ± 13.2	81.6 ± 10.9
Eotaxin-3	0.6 ± 0.1	3.7 ± 0.1	1.4 ± 0.1	1.1	19.6 ± 5.6	138 ± 1.8	0.1
FGF-2	1 ± 0.7	75.1 ± 2.2	6 ± 1.2	7.3 ± 0.9	-	1084.2 ± 28.6	-
Fractalkine	-	-	26.9 ± 0.5	15.9	18.8 ± 4	39.8 ± 1	-
G-CSF	10.5 ± 0.8	53.5 ± 3.1	-	25 ± 1.8	-	21.8 ± 14.2	-
GM-CSF	-	-	3.1 ± 1.5	5.9 ± 3	-	46.4 ± 27.4	-
Gro alpha	1.5 ± 0.2	9.2 ± 0.3	3.6 ± 0.1	3.3 ± 0.1	16.9 ± 1.9	322.1 ± 6.4	-
HGF	-	1.5 ± 1	13.1 ± 1	10.4 ± 0.7	699.6 ± 9.5	272 ± 2	101.2 ± 7.6
IFN-alpha	1.4 ± 0.1	2.2 ± 0.2	1.8 ± 0.2	1.5 ± 0.2	1.5 ± 1.5	5.4 ± 1	-
IFN-gamma	1.6 ± 0.2	1.9 ± 0.3	3.2 ± 0.5	3.2 ± 0.3	18.8 ± 2.1	21.4 ± 3.9	13.2 ± 0.7
IL-1 alpha	-	4.8 ± 0.2	2.4 ± 0.3	1.3 ± 0.5	14 ± 4	75.7 ± 2.2	-
IL-1 beta	3.4 ± 0.9	6.2 ± 0.9	17 ± 1	23.8 ± 3.3	50.2 ± 8.5	224.6 ± 9.6	17.1 ± 4.4
IL-10	-	2.2 ± 0.1	1.4 ± 0.4	1 ± 0.3	-	-	-
IL-12p70	-	0.3 ± 0.1	0.4 ± 0.1	0.5 ± 0.1	3.2 ± 0.4	11.9 ± 1.3	0.1
IL-13	-	-	-	-	0.6 ± 0.6	4.6 ± 1.4	-
IL-15	-	3.3 ± 0.1	0.6 ± 0.3	0.6 ± 0.3	5.1 ± 5.1	33.2 ± 3.2	-
IL-16	-	6.3 ± 2.6	1.9 ± 1.8	10.8 ± 1.9	191.7 ± 38.6	253.1 ± 19.7	909.3 ± 16
IL-17A	-	-	1.3 ± 0.5	1.5 ± 0.8	8.7 ± 5.6	27.6 ± 6.1	-
IL-18	1.5 ± 0.3	2.1 ± 0.2	4.3 ± 0.1	3.2 ± 0.1	16.8 ± 2.3	30.6 ± 2.9	16.1 ± 1.8
IL-2	-	0.2 ± 0.1	0.1 ± 0.1	0.2 ± 0.1	61.3 ± 14.3	177.8 ± 10.5	-
IL-20	2.2 ± 0.1	2.4 ± 0.1	1.4 ± 0.1	1.4 ± 0.1	87.1 ± 7.4	152.9 ± 3.9	-
IL-21	0.5 ± 0.5	6 ± 1.6	3.1 ± 0.4	2.3 ± 0.4	103.3 ± 9.1	245.6 ± 7	-
IL-22	0.3 ± 0.1	17.6 ± 2.2	7.1 ± 0.5	7.1 ± 1	171.3 ± 15.5	356 ± 18.5	-
IL-23	-	221.1 ± 5.5	2.4 ± 2.4	1.7 ± 1.3	-	5978.3 ± 183.8	-
IL-27	-	-	-	-	-	24.7 ± 16.7	-
IL-2R	-	415.5 ± 50	28.7 ± 19.9	44.7 ± 22.3	-	1260.9 ± 377.2	2585.1 ± 300.7
IL-3	-	-	-	-	96.9 ± 46.8	511.3 ± 32	-

IL-31	-	7 ± 2.7	-	8.3 ± 3.9	-	2582 ± 31.9	-
IL-4	12.4 ± 5.1	44.9 ± 2	26.9 ± 2.7	31.3 ± 2.4	-	375.9 ± 26.1	-
IL-5	0.6 ± 0.6	1.6 ± 0.1	-	0.1 ± 0.1	-	-	-
IL-6	-	-	-	-	-	-	-
IL-7	-	-	0.5	0.8 ± 0.1	1.9 ± 0.3	0.6 ± 0.2	-
IL-8	-	1.2 ± 0.3	0.2 ± 0.1	0.3 ± 0.2	-	240.6 ± 11.8	-
IL-9	-	4.2 ± 1.6	-	0.2 ± 0.2	-	262.1 ± 34.4	-
IP-10	1.4	-	1.5	1.7 ± 0.1	211 ± 5.2	75.3 ± 1.3	28.5 ± 2.1
I-TAC	-	-	-	-	70.1 ± 28.7	82.2 ± 3.4	-
LIF	-	-	-	0.2 ± 0.1	0.2 ± 0.1	29.9 ± 1.9	-
MCP-1	-	-	0.3 ± 0.2	-	125.8 ± 1.9	90.7 ± 2.8	75.6 ± 5.1
MCP-2	-	-	0.2	0.1	39.4 ± 2.3	17.6 ± 0.3	6.4 ± 0.5
MCP-3	-	9.2 ± 1.3	9.1 ± 0.6	7.7 ± 0.9	-	-	-
M-CSF	1.1 ± 0.5	38.6 ± 1.8	74.4 ± 3.8	10.7 ± 0.4	12.4 ± 8.7	4341.9 ± 136.9	-
MDC	5.8 ± 1.3	13.1 ± 0.4	7.8 ± 1.2	8.5 ± 0.5	1070.3 ± 5.7	554.8 ± 6.8	139.3 ± 13.3
MIF	0.6	5.9 ± 0.1	9.1 ± 0.3	5.9 ± 0.1	167.2 ± 5	262.6 ± 27.2	119.4 ± 5.6
MIG	-	2.7 ± 1.1	0.3 ± 0.3	-	140.7 ± 45.6	286.4 ± 4.2	-
MIP-1 alpha	-	-	-	-	42.4 ± 3.5	62.5 ± 1.4	-
MIP-1 beta	-	-	-	-	559.2 ± 44.2	716.5 ± 1.2	-
MIP-3 alpha	3 ± 0.3	5.8 ± 0.3	4.2 ± 0.3	4.3 ± 0.2	121 ± 3.2	32 ± 1.2	60.3 ± 2.2
MMP-1	-	0.2 ± 0.1	6.7 ± 0.1	0.3 ± 0.1	4178.9 ± 11.1	4.8 ± 0.4	1853.7 ± 41.2
NGF beta	-	6.6 ± 2.4	-	0.8 ± 0.8	-	26.8 ± 2.4	-
SCF	-	0.3 ± 0.3	5.7 ± 0.4	1.2 ± 0.2	18.7 ± 0.8	16.7 ± 2.2	55.1 ± 1.1
SDF-1 alpha	5.3 ± 5.3	70.7 ± 3.4	81.1 ± 7.6	42.3 ± 1.7	3796.6 ± 93.6	2245.6 ± 54.8	1604.3 ± 25.2
TNF alpha	1.8 ± 0.2	2.8 ± 0.1	2.2 ± 0.1	2.2 ± 0.1	19.8 ± 2.2	33.2 ± 0.9	13.2 ± 1.6
TNF beta	0.3 ± 0.3	4.6 ± 0.4	3.4 ± 0.5	1.6 ± 0.1	-	1566.9 ± 136.4	-
TNF-R2	0.2 ± 0.2	4.9 ± 0.3	3.9 ± 0.5	1.8 ± 0.2	119.6 ± 9	147 ± 2	153.3 ± 10.5
TRAIL	1.8 ± 0.9	8 ± 0.5	4 ± 0.6	2.6 ± 0.6	148.9 ± 13.7	332.6 ± 7.9	-
TSLP	-	0.5 ± 0.2	2.3 ± 0.2	2.5 ± 0.5	24.2 ± 3.2	37.8 ± 2.5	0.5 ± 0.5
TWEAK	-	136.7 ± 6.4	738.6 ± 33.4	588.5 ± 14.2	6676.1 ± 761.7	7403.9 ± 80.3	168.8 ± 94.8
VEGF-A	-	24.3 ± 1.9	33.9 ± 2.3	16.8 ± 0.3	768.3 ± 62.3	1009.2 ± 30.7	22.7 ± 10.5

Supplementary Table 2. Full name for each target in the ProcartaPlex Immune Monitoring 65-Plex Panel

Analyte	Gene name (HGNC)	Protein name (Uniprot)
APRIL	TNFSF13	TNF ligand superfamily member 13, A proliferation-inducing ligand
BAFF	TNFSF13B	TNF ligand superfamily member 13B, B-cell activating factor
BLC	CXCL13	C-X-C motif chemokine 13, B lymphocyte chemoattractant
CD30	TNFRSF8	Tumor necrosis factor receptor superfamily member 8
CD40L	CD40LG	CD40 ligand
ENA-78	CXCL5	C-X-C motif chemokine 5, epithelial-derived neutrophil-activating peptide 78
Eotaxin	CCL11	Eotaxin, C-C motif chemokine 11
Eotaxin-2	CCL24	C-C motif chemokine 24
Eotaxin-3	CCL26	C-C motif chemokine 26
FGF-2	FGF2	Fibroblast growth factor 2
Fractalkine	CX3CL1	Fractalkine, C-X3-C motif chemokine ligand 1
G-CSF	CSF3	Granulocyte colony-stimulating factor
GM-CSF	CSF2	Granulocyte-macrophage colony-stimulating factor, Colony stimulating factor 3
GRO-alpha	CXCL1	Growth-regulated alpha protein, C-X-C motif chemokine ligand 1
HGF	HGF	Hepatocyte growth factor
IFN-alpha		Interferon alpha
IFN-gamma	IFNG	Interferon gamma
IL-1 alpha	IL1A	Interleukin-1 alpha
IL-1 beta	IL1B	Interleukin-1 beta
IL-10	IL10	Interleukin-10
IL-12p70		Interleukin 12p70
IL-13	IL13	Interleukin-13
IL-15	IL15	Interleukin-15
IL-16	IL16	Pro-interleukin-16
IL-17A	IL17A	Interleukin-17A
IL-18	IL18	Interleukin-18
IL-2	IL2	Interleukin-2
IL-20	IL20	Interleukin-20
IL-21	IL21	Interleukin-21
IL-22	IL22	Interleukin-22
IL-23		Interleukin-23
IL-27		Interleukin-27
IL-2R		Interleukin-2 receptor
IL-3	IL3	Interleukin-3
IL-31	IL31	Interleukin-31
IL-4	IL4	Interleukin-4
IL-5	IL5	Interleukin-5

IL-6	IL6	Interleukin-6
IL-7	IL7	Interleukin-7
IL-8	CXCL8	Interleukin-8
IL-9	IL9	Interleukin-9
IP-10	CXCL10	C-X-C motif chemokine 10, Interferon gamma-induced protein 10
I-TAC	CXCL11	C-X-C motif chemokine 11, Interferon-inducible T-cell alpha chemoattractant
LIF	LIF	Leukemia inhibitory factor
MCP-1	CCL2	C-C motif chemokine 2, Monocyte chemoattractant protein 1
MCP-2	CCL8	C-C motif chemokine 8, Monocyte chemoattractant protein 2
MCP-3	CCL7	C-C motif chemokine 7, Monocyte chemoattractant protein 3
M-CSF	CSF1	Macrophage colony-stimulating factor 1
MDC	CCL22	C-C motif chemokine 22, Macrophage-derived chemokine
MIF	MIF	Macrophage migration inhibitory factor
MIG	CXCL9	C-X-C motif chemokine 9, Monokine induced by gamma interferon
MIP-1 alpha	CCL3	C-C motif chemokine 3, Macrophage inflammatory protein 1-alpha
MIP-1 beta	CCL4	C-C motif chemokine 4, Macrophage inflammatory protein 1-beta
MIP-3 alpha	CCL20	C-C motif chemokine 20, Macrophage inflammatory protein 3 alpha
MMP-1	MMP1	Interstitial collagenase, Matrix metallopeptidase 1
NGF beta	NGF	Beta-nerve growth factor
SCF	KITLG	Kit ligand, Stem cell factor
SDF-1 alpha	CXCL12	stromal cell-derived factor 1 alpha
TNF alpha	TNF	Tumor necrosis factor
TNF beta	LTA	Lymphotoxin-alpha
TNF-R2	TNFRSF1B	TNF receptor superfamily member 1B
TRAIL	TNFSF10	TNF ligand superfamily member 10, TNF-related apoptosis-inducing ligand
TSLP	TSLP	Thymic stromal lymphopoietin
TWEAK	TNFSF12	TNF ligand superfamily member 12, TNF-related weak inducer of apoptosis
VEGF-A	VEGFA	Vascular endothelial growth factor A

Full Western Blot Images from Figures. The red enclosed section was used for Figure 2d.

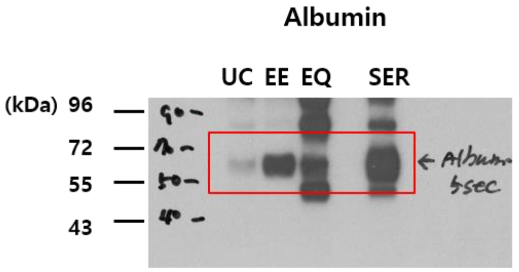

Albumin Ab: Cell Signaling #4929
Molecular Weight: 67kDa

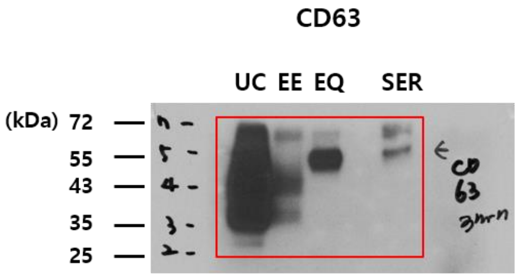

CD63 Ab: SantaCruz sc-5275
Molecular Weight of CD63 core protein: 26 kDa.
Molecular Weight of glycosylated CD63: 30-60 kDa.
